# The evolution of myocardium at risk by T2-STIR MR imaging the first week after acute myocardial ischemia

**DOI:** 10.1186/1532-429X-18-S1-P94

**Published:** 2016-01-27

**Authors:** David Nordlund, Gert Klug, Einar Heiberg, Sasha Koul, Terje H Larsen, Pavel Hoffmann, Bernhard Metzler, David Erlinge, Dan Atar, Anthony H Aletras, Marcus Carlsson, Henrik Engblom, Håkan Arheden

**Affiliations:** 1 Department of Clinical Physiology, Clinical Sciences, Lund University, Lund, Sweden; 2 University Clinic of Internal Medicine III, Cardiology and Angiology, Medical University of Innsbruck, Innsbruck, Austria; 3 Department of Biomedical Engineering, Faculty of Engineering, Lund University, Lund, Sweden; 4 Department of Cardiology, Clinical Sciences, Lund University, Lund, Sweden; 5 Department of Heart Disease, Haukeland University Hospital, Bergen, Norway; 6 Department of Biomedicine, University of Bergen, Bergen, Norway; 7 Department of Cardiology B, Oslo University Hospital Ullevål and University of Oslo, Oslo, Norway; 8 Laboratory of Medical Informatics, School of Medicine, Aristotle University of Thessaloniki, Thessaloniki, Greece; 9 Section for Interventional Cardiology, Department of Cardiology, Oslo University Hospital, Ullevål, Oslo Norway

## Background

Myocardial salvage is currently being used as endpoint in several clinical trials and is determined by relating final infarct size to myocardium at risk (MaR). T2-weighted imaging (T2-STIR) cardiac magnetic resonance (CMR) has previously been shown to enable assessment of MaR up to one week after acute myocardial infarction. Recent experimental data indicate that the extent of MaR by T2-STIR varies over the first week which would have implications on how to design clinical cardioprotection trials using myocardial salvage as endpoint and in the clinical diagnosis of patients with myocardial infarction and normal coronary arteries.

To investigate whether MaR as assessed by T2-STIR differs depending on scan day during the first week after the acute event in patients with reperfused first-time myocardial infarction.

## Methods

196 STEMI-patients from the MITOCARE and CHILL-MI trials undergoing acute percutaneous coronary intervention were included in the study. Eight additional patients with CMR on day 1 were also included. T2-STIR MR imaging was performed 1-7 days after the acute event and was used to evaluate MaR. Diagnostic quality on a scale from 0-3 and ability to correctly assign culprit vessel compared to angiography.

## Results

**Figure 1:**
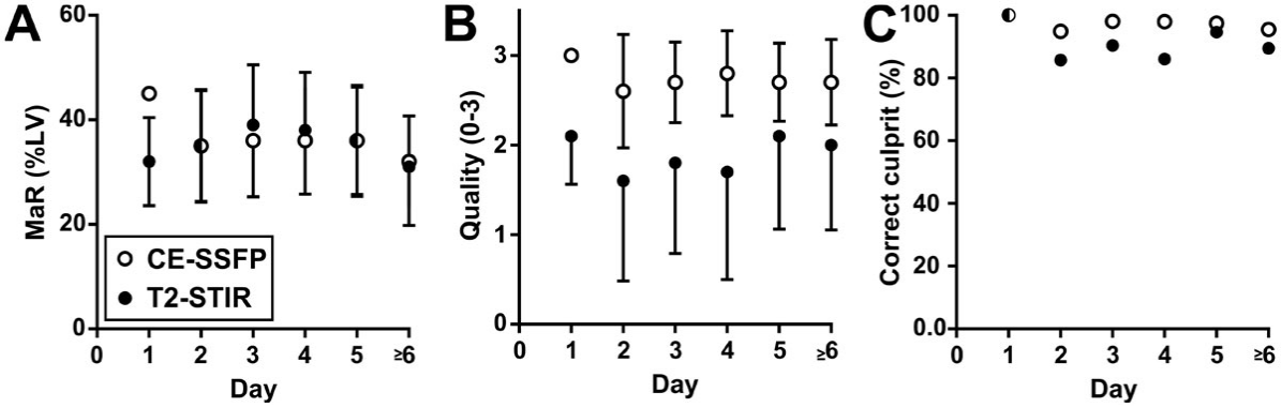
**MaR by T2-STIR over the first week**. A) MaR as % of LV mass over time; B) diagnostic image quality over time; and C) Ratio of correctly assigned culprit vessel compared to angiography over time. Note that there is no significant change in MaR during the first week in any of the above aspects. Open and closed circles show mean values of MaR by CE-SSFP and T2-STIR respectively. Error bars show 1 standard deviation. MaR=Myocardium at risk, LV=Left ventricular mass, T2-STIR= T2-weighted short tau inversion recovery, CE-SSFP = contrast enhanced steady state free precession, LGE=late gadolinium enhancement.

There was no significant difference in MaR over the first week (p = 0.44, Figure 1A) neither was there any change in diagnostic quality (p = 0.26, Figure 1B). The rate of correctly assigned culprit vessel was also similar for the different scan days (Figure 1C).

## Conclusions

Myocardium at risk by T2-STIR CMR imaging do not change in humans over the first week after acute reperfused myocardial infarction suggesting that MaR is a stable measure during this period of time.

